# Cognitive Maps for a Non-Euclidean Environment: Path Integration and Spatial Memory on a Sphere

**DOI:** 10.1177/09567976241279291

**Published:** 2024-10-25

**Authors:** Misun Kim, Christian F. Doeller

**Affiliations:** 1 Max Planck Institute for Human Cognitive and Brain Sciences, Leipzig, Germany; 2 Kavli Institute for Systems Neuroscience, Trondheim, Norway; 3 Wilhelm Wundt Institute of Psychology, Leipzig University; 4 Department of Psychology, Technical University Dresden; 5 Institute of Cognitive Neuroscience, University College London

**Keywords:** spherical geometry, path integration, Euclidean, non-Euclidean, cognitive map, open data, open materials, preregistered

## Abstract

Humans build mental models of the world and utilize them for various cognitive tasks. The exact form of cognitive maps is not fully understood, especially for novel and complex environments beyond the flat Euclidean environment. To address this gap, we investigated *path integration*—a critical process underlying cognitive mapping—and spatial-memory capacity on the spherical (non-Euclidean) and planar (Euclidean) environments in young healthy adults (*N* = 20) using immersive virtual reality. We observed a strong Euclidean bias during the path-integration task on the spherical surface, even among participants who possessed knowledge of non-Euclidean geometry. Notably, despite this bias, participants demonstrated reasonable navigation ability on the sphere. This observation and simulation suggest that humans navigate nonflat surfaces by constructing locally confined Euclidean maps and flexibly combining them. This insight sheds light on potential neural mechanisms and behavioral strategies for solving complex cognitive tasks.

## Introduction

Humans build a mental model or a cognitive map of the environment to efficiently navigate within it. A cognitive map is a useful framework that can describe many cognitive processes beyond physical navigation, such as categorization, concept learning, and planning (Behrens et al., 2018; Bellmund et al., 2018; Gärdenfors, 2000; O’Keefe & Nadel, 1978). Exactly how humans develop cognitive maps and which form they take (e.g., metric, graph, hybrid format) are essential questions in cognitive science that still lack a definite answer.

**Fig. 1.**
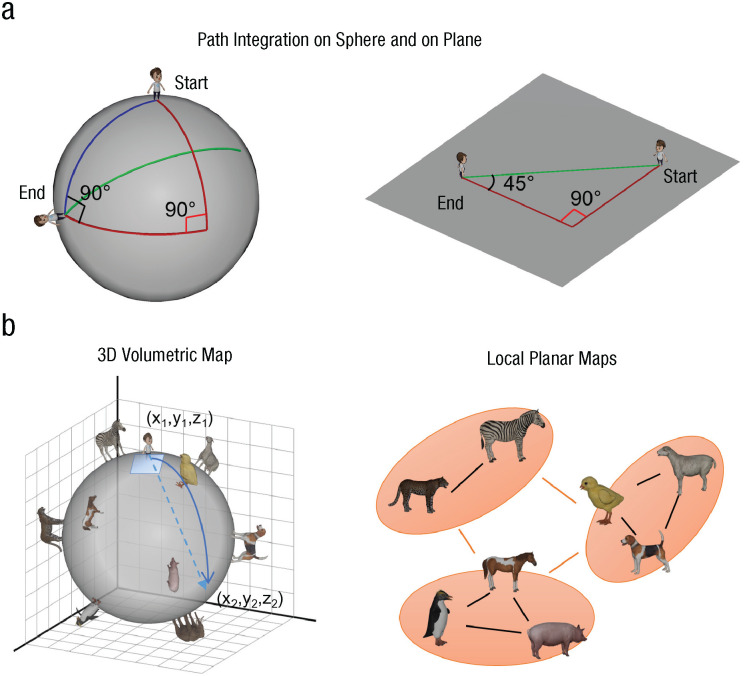
Path integration and cognitive maps for a spherical and planar surface. If people rely on a path-integration mechanism built on a flat surface (a), they will make a systematic error (overturn and overshoot) on the sphere. Red lines represent the outbound journey; green lines represent the inbound journey for planar geometry; and blue lines represent the inbound journey for spherical geometry. Spatial layout on the spherical surface (b) can be encoded in a global 3D map or multiple patches of planar maps.

When we explore our environment, we sequentially move from one location to another. Path integration is the ability to keep track of one’s location by integrating previous movements. Path integration serves as a basis for building cognitive maps, and it is universally observed from insects to mammals (McNaughton et al., 2006; Wang, 2016). Importantly, the neural circuitry for path integration (e.g., head-direction cells, grid cells) has mainly been studied when animals navigate on flat 2D surfaces, except for a few recent studies (Ginosar et al., 2021; Grieves et al., 2020; Ulsaker-Janke et al., 2023). For accurate navigation in 3D space with undulating terrain, one needs to integrate 3D movement directions and build a global 3D map. However, building a 3D map can be costly, and the neural circuitry for path integration may be optimized for 2D Euclidean geometry, particularly for surface-dwelling animals like humans. Nonetheless, relying on 2D path integration would result in systematic errors when people try to navigate a nonflat surface like a sphere. For instance, if people move straight from the North Pole to the equator, turn 90 degrees to the right, and then move the same distance (see the red line in Fig. 1a), they will be on the equator. They could then turn 90 degrees again and move straight back to the North Pole (see the blue line in Fig. 1a). However, a path-integration system, optimized for a flat surface where the sum of angles of a triangle is 180 degrees, will tell the agents to turn with an inner angle of 45 degrees (see the green line in Fig. 1a).

**Statement of Relevance**

Humans build mental models of their surroundings. The mental models are called *cognitive maps*, and they help us navigate the world. The neural underpinnings of such maps, and how humans build on them, have primarily been studied in flat 2D worlds. Little is known about how the process generalizes to more complex, high-dimensional environments. Here, using virtual reality, we assessed how people form a map and navigate in a spherical environment. Participants walked on a small planet (as in *The Little Prince*) and searched for the optimal path on this curved surface. We found a strong Euclidean bias that leads to systematic errors on the sphere in all participants. Our results suggest that humans build multiple local planar maps to navigate in a complex environment by utilizing neural circuitry optimized for 2D navigation. This strategy might be useful for more general cognitive tasks beyond physical navigation.

If the path-integration system is predominantly 2D, people are likely to build multiple local planar maps that cover the curved surface (Fig. 1b), rather than building a global 3D map (Fig. 1b). Path planning would be particularly difficult for long distances on a sphere if participants use local planar maps, because planning a route across multiple regions could be more difficult than planning within a region (Montello & Pick, 1993; Wiener & Mallot, 2003). In contrast, agents using a global 3D metric map should be able to draw a straight 3D shortcut and project it to the surface to find the optimal path, regardless of distance.

In this preregistered study we built spherical and planar virtual environments tightly matched in visual appearance and complexity. In an immersive virtual-reality (VR) setting, participants performed a path-integration task known as a *triangle completion* (Chrastil & Warren, 2021) and spatial-memory tests with landmarks. We examined whether participants relied on 2D path integration, consequently using planar maps instead of a global 3D map. We then simulated the efficacy of multiple planar maps for solving a navigation problem on a spherical surface.

## Method

### Participants

Twenty healthy young participants completed the experiment (10 male, mean age = 26.4 years, *SD* = 4.8 years). This study was approved by a local ethics committee.

### Justification for sample size

We determined this sample size on the basis of the pilot data, which are reported in preregistration (https://osf.io/hv29w). For the triangle-completion task, we generated 1,000 data sets for the turn angle and distance using either planar or spherical geometry prediction using the *Mixedpower* R package (Kreidler et al., 2021; Kumle et al., 2021). We then counted the number of simulations when the approximated Bayes factor (BF) for the true geometrical model was greater than another model by 10. With a sample size of 20, the power was 100%. For the comparison of short and long trial errors in the object-location memory test, we used the Bayes factor design analysis R package to simulate the paired *t* test result for the effect size estimated from the pilot data (*d* = 1.9; Stefan et al., 2019). Simulation (1,000 iterations) with a sample size of 20 predicted that 100% would reach the sensitivity threshold (BF = 3) and a false positive rate was less than 1%.

### Virtual environment and equipment

**Fig. 2.**
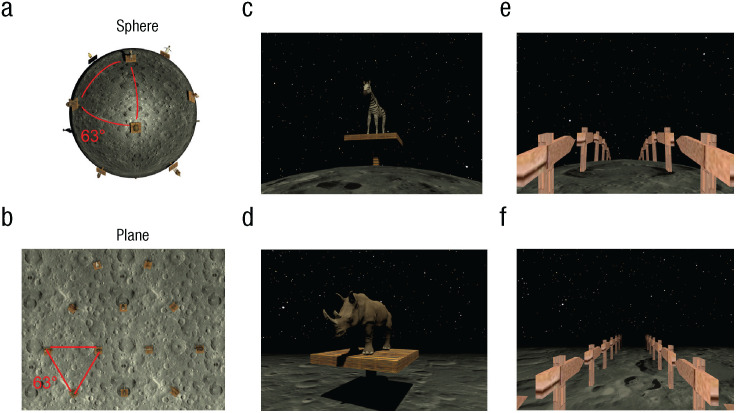
Virtual environments. Bird’s-eye views of the sphere and planar environments are shown in (a) and (b), respectively. Both environments featured the same moon-like texture and a dark background. Twelve animal landmarks were positioned in a regular triangular lattice with the geodesic distance between the neighboring landmarks identical in the two environments. In (c) and (d) are example views from a first-person perspective during the object-location task on the sphere and planar surfaces. Visibility was matched in the two environments thanks to the fog. In (e) and (f) are shown example views of the corridor that participants walked along during the triangle-completion task. Of note, participants performed the tasks with a VR headset, and the field of view and depth perception were of higher quality than the current snapshots.

We used Unity 2020.2.6.f1 (Unity Technologies, San Francisco, US) to implement the two virtual environments that contain either a planar or spherical surface (Fig. 2; see also Supplemental Videos S1 and S2 in the Supplemental Material available online). Participants walked on an omnidirectional treadmill (Virtualizer 2, Cyberith GmbH, Vienna, Austria) while wearing a head-mounted display (Valve Index, Valve, Bellevue, US) to explore the virtual environments. Both environments had a moon-like appearance and the same starry sky as a background. The textured surface and sky provided optic flow and a sense of motion to participants. The radius of the sphere was 12.5 virtual units, and the camera height was 1.5 virtual units. On the sphere, arc lengths presented in degrees gave a more intuitive sense of the distance than the distance presented in the virtual units. For example, it is easier to know that 180° corresponds to half of the circumference, as opposed to 39.2 virtual units. Thus, we used degrees as the unit to describe both length and angular variables in this article. Maximum translational speed was 20°/s so that participants could complete the circle in about 18 s if they walked without stopping.

For the object-location tasks, we placed a unique set of 12 animals on the planar and spherical surfaces. On the plane, the animals were located at the vertices of triangular tessellation, and all faced toward a fixed point near the center (Fig. 2b). On the sphere, the animals were placed at the 12 vertices of a regular icosahedron, which corresponds to a triangular tessellation of a spherical surface. The animals faced toward the imaginary north pole of the sphere (Fig. 2a). Participants were explicitly told that they could rely on the fixed-point-facing animals to get oriented. These animals served as both local landmarks and global-orientation cues (e.g., an imaginary north-south axis). The geodesic distance between the neighboring animals on the plane and sphere was identical (63°).

Importantly, we also matched the visible distance of the two environments. In an open field, people can normally see things on the plane that are very far away from them. In contrast, people cannot see things that are positioned far away on the curved surface because the light travels straight and is obstructed by the surface itself. By adding fog, we matched the number of visible landmarks in the two environments.

### Experiment

#### Task overview

Participants came to the laboratory on two separate days. Half were tested in the spherical environment on the first day and the plane on the second day, and the other half did the reverse. On the first day, via a desktop computer, participants were given detailed instructions about all the upcoming tasks. They then had a short movement-practice session on the VR treadmill before starting the main experiment. We warned participants of potential VR-related motion sickness and offered ginger tea and candies that might alleviate motion-related nausea. The experiment stopped immediately if the participant felt uncomfortable. On each day, the same set of primary tasks was completed in the following order: learning by free exploration, object-location memory training, object-location memory test, triangle-completion tasks, and debriefing. In total, participants spent 2.5 to 3 hrs per day. To increase the motivation of participants, we offered a small monetary bonus at the end of the experiment, depending on the spatial-memory performance.

#### Movement practice on the VR treadmill

The VR treadmill had optic sensors on the platform that recorded the movement of participants’ feet. A rotatable ring, at waist level, recorded any change in direction. Participants walked forward and rotated their bodies to change the direction of movement. Backward walking was not supported. When they moved “straight” on the sphere they followed a great circle such that they would return to where they started after walking for a while. While practicing the movement for a few minutes, participants collected virtual traffic cones placed on the surface.

#### Learning by free exploration

Participants were given 15 min to freely explore the environment and learn the location of the 12 animals as precisely as they could. A list of all animals was shown at the top of the screen. This allowed participants to easily check whether they had found each animal in the foggy environments. On the plane, participants heard an alarm if they reached an invisible boundary of the environment. Participants were instructed to return to the center of the plane, where the animals were located, if they hear the alarm. On the sphere such an alarm was unnecessary, because the sphere does not have a boundary, and participants could always find animals in any direction. The task is shown in Supplemental Videos S1 (https://osf.io/2es6n) and S2 (https://osf.io/jpm5a), and the actual trajectories of participants are shown in Supplemental Figures S1 and S2. Participants tended to repeatedly walk along a great circle on a sphere, whereas the exploration pattern on a plane was more variable across participants.

#### Object-location memory training and test

After the initial learning task, participants were asked to find target animals from pseudorandom starting locations. At the beginning of each trial, a target animal was cued with a picture, and then participants had to indicate which direction would lead to the shortest path to the target. Once they had indicated the optimal direction with a button press, they could move freely to find the target animal. All animals, including the target, were present in the environment, so participants were able to change their trajectory after seeing other animals while on the move. Once they found the target, a corridor that linked the start and target location by the shortest path was shown. This corridor taught participants about the optimal route on the sphere. Thus, we gave participants a fair opportunity to learn the spatial layout and to practice route planning in the unfamiliar spherical condition. Participants completed two runs of 12 training trials.

After the training, participants were given a similar target-searching task. This time, animals were visible only at the start, and the rest of the animals, including the target, were hidden after the participant began to move. This meant that participants had to accurately estimate the distance and direction to the target at the start of the trial and try to move straight thereafter. During movement they had to update their location on their mental map by integrating motion cues (e.g., proprioception, optic flow from the floor texture and starry sky, and the passage of time). After participants indicated the location of the hidden target with a button press, they received feedback on their performance, measured by the geodesic distance between the true location and indicated locations (on a 5-point smiley-face scale ranging from a frowning face to a smiling face). Additionally, the optimal route was shown during feedback.

Critically, we varied the distance between the starting location and the target location to test whether path planning was more difficult, particularly for long-distance trials on the sphere. In half of the trials, targets were directly behind visible landmarks (short trials), whereas extra hidden landmarks were between the start and the target in the other half (long trials). Unbeknownst to participants, only 6 of the 12 animals were used as targets. This was done to increase the number of repetitions per target object within the limited experiment duration. Because of the random starting location, a good knowledge of the layout of all 12 animals was required to perform well in this task. Each of the six target objects was presented twice in each run, and each participant completed two runs. The task is shown in Supplemental Videos S3 (https://osf.io/aux7s) and S4 (https://osf.io/nw3mc).

#### Triangle-completion test

Last, participants were given a commonly used path-integration task called the *triangle-completion task* (Chrastil & Warren, 2021). During this task, all animal landmarks were removed, and participants saw only a narrow corridor consisting of two straight legs (Figs. 2e and 2f). Participants first walked straight until the end of the first leg of the corridor. Then the second leg of the corridor became visible, and participants turned and kept walking until the end of it. Upon arrival, they first indicated the optimal direction back to the starting location. Subsequently, they walked straight to the remembered starting location. During the response phase, the corridor was invisible so that participants could not rely on visual traces to find the starting location.

To motivate the participants, we gave feedback on the geodesic-distance error between the true starting location and their response location, using a 5-point smiley-face scale ranging from frowning to smiling. However, we kept the corridor invisible during feedback. Therefore, it was not possible for participants to notice if they made a systematic error (e.g., overturning on the sphere) and simply adjust their response strategy. In this way, we could probe their pure path-integration ability and their sense of geometry on the novel spherical and planar surfaces.

We used seven triangle shapes (see Supplemental Fig. S3). We carefully selected the triangle-shape parameters to have a large range of inbound turn angle and distance. This was to prevent participants from following a stereotypical response that was insensitive to the exact shape of the triangles. Insensitive responses were a concern in a previous study (Kearns et al., 2002). The shape parameters—the lengths of the two legs and the angle between them—were identical for the planar and spherical conditions, but the ideal inbound turn angle and length were different because of geometry. This allowed us to test whether participants’ responses were closer to the planar or spherical solution. We presented each triangle shape four times across the two runs. Each triangle shape was presented with left/right symmetry; that means that participants turned left on half of the trials and turned right on the other half of the trials. The trial order was randomized. The task is shown in Supplemental Videos S5 (https://osf.io/ks639) and S6 (https://osf.io/y75nu).

#### Debriefing

Each day, at the end of the experiment, we asked participants about their strategy (e.g., first-person, bird’s-eye view) for each task. For the triangle-completion task on the sphere, we asked whether they had thought about how much they moved on the sphere, in terms of absolute length (such as 90°). We also asked whether participants had any knowledge about the difference between planar and spherical geometry (e.g., that the sum of the angles of a triangle is not 180° on the sphere).

### Analysis

#### Triangle-completion task: main hypothesis testing

We examined whether participants used planar or 3D path integration on the sphere using two types of measurements: inbound turn angle and distance. As a preprocessing step, the left and right symmetric triangles were pooled. This restricted the ideal turn angle to a range of 0° to 180°, rather than −180° to 180°. Thus, we could treat the turn angle like a linear variable. Previous studies have also treated the turn angle as a linear variable by following the same procedure (Chrastil & Warren, 2021; Kearns et al., 2002; Tcheang et al., 2011). We defined trials as outliers when either the inbound turn angle or distance was outside 1.5 times the interquartile range of all participants’ data, within each triangle type. Outlier trials were excluded from further analysis (52 out of 1,119 trials in total). We built two simple linear mixed models with the main predictors being, respectively, the ideal turn angle by planar or spherical geometry. All participants’ turn angles were the dependent variable. Given that each participant could have shown different sensitivity to the ideal turn angle, as well as a different intercept, we included the random intercept and slope for each participant. We then compared whether the spherical or planar model fitted the data better by comparing the associated Bayesian information criteria (BICs). The BF was approximated as exp(difference in BIC/2) (Raftery, 1999). A large BF for the planar model (BF > 3) would suggest that people rely on a 2D path-integration system, even when they are on the sphere. In contrast, a small BF (BF < 1/3) would suggest that people can use a volumetric map and 3D path-integration system on the sphere. In addition to the BF for the linear mixed model, we report the mean *R*^2^ for the linear model of each participant. This can help readers understand how well the planar and spherical geometry models fitted the actual data at the individual participant level. We repeated the analysis process for the inbound distance variable. As a quality check, we also ran the same analysis for the data from the plane condition, in which the planar-geometry model should obviously fit the data better.

#### Triangle-completion task: additional analysis

In addition to the preregistered main hypothesis testing, we conducted the following exploratory analyses. First, we built a model that contains both planar and spherical predictors and tested whether this combined model could explain the data better than the planar model alone, using the BIC. Second, we constructed spherical models with various radii, ranging from 1 to 3 times the original radius, to determine the best-fitting radius (Supplemental Fig. S4). We then evaluated whether the larger-radius spherical model provided a better fit to the actual data than the planar model at both the group and single-subject levels. A radius three times the original was large enough to make the spherical model’s predictions indistinguishable from those of the planar model, eliminating the need to simulate larger radii. At both group level and single-subject level, we checked whether the larger-radius spherical model could fit the actual data better than the planar model. Third, we tested whether participants may have tried to adjust their path-integration system to the curved surface by using more cognitive resources (through an analysis of reaction time) or a bird’s-eye-view perspective, compared to when they were doing the same task in the plane condition. We performed a *t* test on the mean reaction time and McNemar’s test on the proportion of participants who used the bird’s-eye-view strategy in the plane and sphere conditions. Last, we divided the participants into two groups on the basis of their self-reported prior knowledge of spherical geometry and tested whether those with geometrical knowledge also showed a planar bias on the sphere.

#### Object-location memory test

Our main hypothesis concerned the optimal route-planning ability that depends on whether participants use a 3D Euclidean map or multiple planar maps. There were two types of errors reflecting the optimal route-planning ability: a direction error, which is an angular deviation of a participant’s initial route from the ideal route to the target, and a position error, which is a geodesic distance between the recalled location and the true location of the target.

As a first step of the analysis, we defined outlying trials (position error beyond 1.5 times the interquartile range within each participant) and removed them. A few large positional errors could occur if participants swapped the identity of landmarks. We then grouped trials into short or long on the basis of the geodesic distance between the start and target locations, with the distance threshold of 110°. Next, the mean direction error for the short and long trials in the plane and sphere conditions was computed for each participant (2 × 2 factors). Long-distance trials could generally be harder (main effect of distance). However, if participants used multiple planar maps instead of building a global 3D map, the direction error was expected to be particularly large for long-distance trials on the sphere (interaction effect). Thus, we tested for this interaction effect using a Bayesian *t* test on the difference between the long- and short-distance trials on the sphere and on the plane. We performed the *t* test using the Bayes factor R package and JASP with the default objective prior (Cauchy distribution with a scale parameter of *r* = $\sqrt{2}/2$ ). We also report the statistics from a classical *t* test for completeness, but our conclusion was based on the BF.

#### Simulation of route planning based on multiple planar maps

To simulate the route-planning performance, we sampled random start locations with varying distance from one fixed target location on the sphere (Supplemental Fig. S7). Because of the rotation symmetry of a sphere, it is sufficient to use one fixed target. A spherical surface could be divided into triangular patches using 12 landmark animals that were evenly distributed on the surface (i.e., vertices of icosahedron). When multiple planar maps are used to approximate the spherical surface, triangular patches could be arranged on a hypothetical plane optimally or suboptimally (see the Results section). Every start location on a spherical surface was projected to a location on a triangular patch. We then computed the direction and distance of a path from each start location to the target on the plane. Next, we drew a path on the sphere using the direction and distance computed on the plane; we then measured angular and location errors on the sphere.

## Results

### Path integration on the sphere is better explained by a planar geometry model

**Fig. 3.**
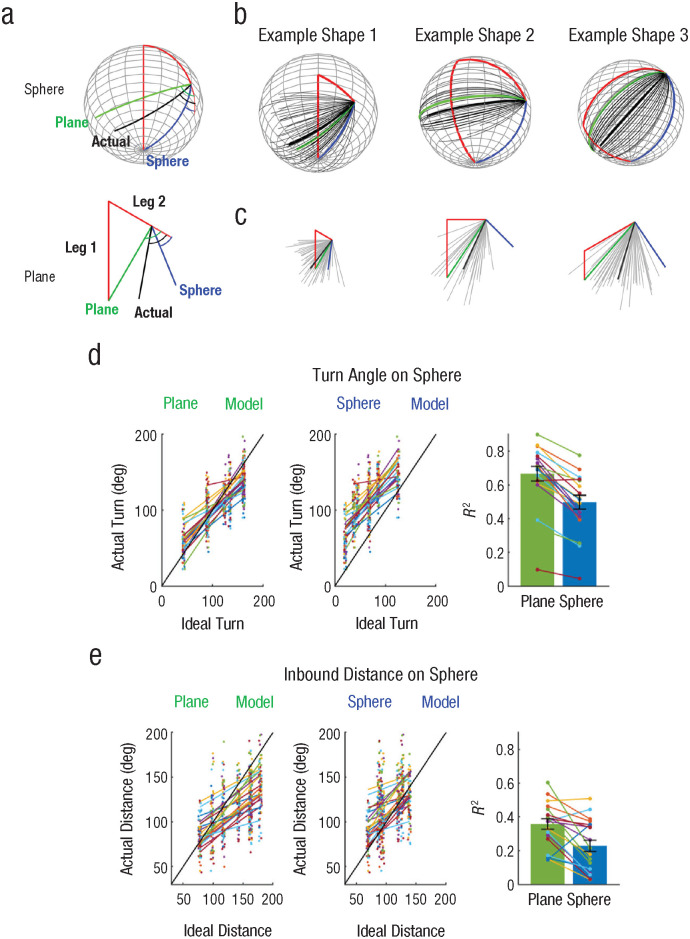
Triangle-completion task. Following an outbound journey consisting of two legs (a; red line), participants turned and moved straight to the invisible start location (black line). Participants performed the same task on the sphere and plane. If a path-integration system is based on planar geometry, participants were expected to follow the green line in both conditions. In contrast, participants would follow an optimal route on the sphere (blue line) if the path-integration system is adjusted for spherical geometry. All participants’ inbound paths on the sphere (b) are shown for three example triangle shapes. Gray lines show individual trials; the thick black line represents the mean path of all trials. In (c) the same is shown for the plane condition. The comparison of two linear mixed models in (d) shows that actual turn angle on the sphere was better explained by the ideal turn angle on the planar model. Individual participant data are colored together, and a small jitter is applied on the horizontal axis for visualization purposes. A bar graph shows the *R*^2^ of model fit for the plane and sphere model at the individual-participant level. Error bars show standard errors. In (e) the same is shown for the inbound distance.

Participants performed a triangle-completion task on both the sphere and plane. Figure 3b and 3c shows the raw data from three example shapes (all shapes are shown in Supplemental Fig. S3). It is apparent that participants’ trajectories (black lines) were closer to the ideal response for planar geometry (green line) than those for spherical geometry (blue line). This was true in both the plane and sphere conditions. A comparison of two linear mixed models revealed strong evidence for the planar-geometry model over the spherical model when people were on the sphere (approximated BF for the planar vs. sphere model: turn angle, BF = 3.5e54; distance, BF = 2.6e23). Supplemental Tables S1 through S4 contain a detailed report on the fixed and random effects for each model. Next, we report how well individual participants’ turn angles and distances were fitted to the ideal turn angle and distance for the planar and spherical geometry. A strong linear relationship between the actual data and predictors were observed in most participants, and *R*^2^ values for individual participants were higher for the plane model than the sphere model for the turn angle (the planar geometry model = 0.67 ± 0.19 vs. sphere model = 0.50 ± 0.18) as well as for distance (the planar geometry model = 0.36 ± 0.13 vs. sphere model = 0.23 ± 0.15; Figs. 3d and 3e). These results imply that participants used a 2D path-integration system rather than a precise 3D path-integration system based on a volumetric map.

### Planar bias prevails: exploratory analyses on mixed models, spherical models of various radii, reaction time, strategy, and previous knowledge of spherical geometry

As exploratory analyses, we tested whether participants tried to overcome the strong planar bias on the sphere. If that was the case, a mixed influence of planar and spherical geometry would be expected. We built a model that contains both planar and spherical geometry predictors and compared it to a model that contains only the planar predictor. The planar-geometry model remained the clear winner over the mixed-geometry model (BF = 1.3e4 for the turn angle, BF = 2.1e3 for distance). We also built the spherical models with various radii to check whether participants overestimated the radius of the sphere and whether a larger-radius spherical model could fit the data better than a plane model. A group-level analysis showed that larger-radius models fitted the data better than smaller-radius models, and the plane model outperformed the spherical model of all radii we tested (Supplemental Fig. S4c). We also investigated the best-fitting radius at the single-subject level. The model with the largest radius was often the best model (Supplemental Fig. S4d). Because of the small number of data points at the single-subject level and reduced model discriminability between larger radii, the best-fitting radius should be interpreted with caution.

Next, we compared the reaction time and self-reported strategy for the path-integration task in the sphere and plane conditions. We reasoned that if participants consider the curvature of the surface and update their location by using a 3D map, reaction time would be slower on the sphere and they would be more likely to use the bird’s-eye view (third-person perspective) than a first-person perspective on the sphere. Neither the reaction time nor the proportion of bird’s-eye-view strategy use was larger for the sphere—mean time on sphere = 6.4 ± 2.5 s versus on plane = 7.0 ± 2.4 s, *t*(19) = 1.1, *p* = .85, one-sided; proportion of bird’s-eye-view perspective on sphere = 7 out of 20 vs. on plane = 5 out of 20, McNemara test, *p* = .31. We also note that none of the participants reported paying attention to the absolute traveled distance relative to the size of the sphere, which was necessary for path integration based on a 3D map.

Finally, we tested whether explicit knowledge of 3D geometry helped people to overcome the planar-geometry bias and to utilize a 3D map. Those who possessed geometrical knowledge of the sphere (*n* = 9) showed a similarly strong planar bias on the sphere, as did those without the knowledge (*n* = 11; Supplemental Fig. S5).

In sum, participants performed the path-integration task on the sphere as if they were still on the plane. Thus, the planar bias cannot be readily eliminated by explicit knowledge of spherical geometry. In the next sections, we present how people with such planar bias performed in the object-location memory task.

### Large direction error at the start, but small position error at the end

**Fig. 4.**
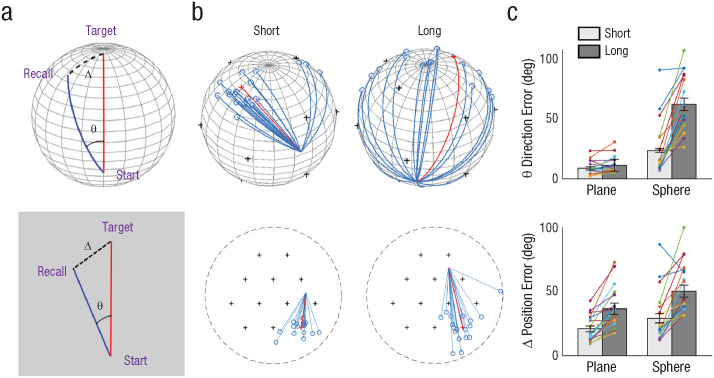
Object-location memory tasks. Two types of error (a) were measured in this task, in which participants had to walk straight to an invisible target from various start locations on a sphere (top) and a plane (bottom). *Direction error* (θ) refers to the angle between the optimal route (red line) and the path taken by participants (blue line). *Position error* (—) refers to the geodesic distance between the true target location and the recalled location (black dotted line). In (b), example optimal paths (red lines) and all participants’ trajectories (blue lines) are shown when the start locations were either at a short or long distance from the target. If participants used multiple planar maps instead of a volumetric map, path planning would be particularly difficult for long trials on the sphere. Indeed, direction errors (c) were significantly larger for long trials on the sphere, and a significant interaction effect between the distance and environment type was observed. Position errors (c) were significantly larger for long-distance trials on both the plane and the sphere, without an interaction effect. Colored dots represent each participant’s mean error; error bars represent standard errors.

When participants rely on planar maps instead of a 3D map, path planning for long-distance trials on the sphere is expected to be particularly challenging. Indeed, we observed a significantly larger initial direction error on the sphere compared to the plane when the target was farther away (Figs. 4b and 4c). The angular difference between long and short trials was 2.5 ± 4.8° on the plane, but a substantial 38.5 ± 19.4° on the sphere—BF = 1.1e5, interaction effect, *t*(19) = 7.7, *p* < .001, Cohen’s *d* = 1.7. We also found that the final position error was larger for long-distance trials on both sphere and plane. However, no significant interaction effect was observed. The difference in error between long and short trials on the plane was 15.8 ± 10.4°, whereas on the sphere it was 21.3 ± 18.3°, BF = 0.8, *t*(19) = 1.3, *p* = .2, Cohen’s *d* = 0.3.

It is worth noting that participants achieved better accuracy than chance even in the long-distance trials on the sphere (mean position error for long trials on sphere = 50.6 ± 20.4°, chance = 90°; final errors for all targets are displayed in Supplemental Fig. S6). This dissociated pattern of error for direction and position was possible because of the nonflat geometry of the sphere, where routes that initially deviate significantly from the shortest path can eventually converge near the target.

### Reasonable navigation performance can be achieved by using multiple planar maps

**Fig. 5.**
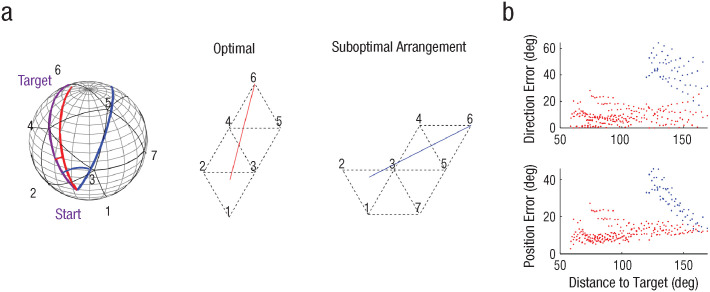
Path planning on the sphere based on multiple planar maps. Local triangular maps (a) are stitched together to find a route to the target. The maps can be aligned either optimally or suboptimally on a hypothetical plane. An example path is shown for a target at position 6 and a start location between positions 1, 2, and 3 (with a distance of 134° to the target). The purple line represents a geodesic line on the sphere; a red line represents a path based on an optimal arrangement of the planar map; and a blue line represents a path based on a suboptimal arrangement. Direction and position errors for trials with varying distance between the start and target are shown in (b), with red dots representing errors based on the optimal arrangement and blue dots representing errors based on the suboptimal arrangement. We simulated the suboptimal arrangement only for distances far enough (> 120°), as it is unlikely that people would recall suboptimal routes when the target is close to the start location.

We conducted simulations to assess the accuracy of path planning on a sphere based on multiple planar maps. In this simulation, we divided the spherical surface into triangular patches that can be dynamically aligned for path planning. In an optimal scenario, participants would recall the minimal number of landmarks between the start and target and align the map accordingly (e.g., if the start is between landmarks 1, 2, and 3, the shortest route to the target, landmark 6, would include landmarks 4 and 5; see Fig. 5a, middle panel). The path direction and distance can then be estimated by drawing a straight line on an imaginary plane and executed on the sphere (see the red line in Fig. 5a). This path, based on the optimally arranged planar maps, closely approximates a true geodesic path on the sphere, resulting in a position and direction error of less than 20° for distant targets (Fig. 5b, red dots). Next, we simulated a scenario in which the agent recalls a suboptimal route and aligns the planar map differently. For example, the agent might first recall landmark 7, followed by 5, 4, and finally the target 6 (see the blue line in Fig. 5a, right side). The direction and position error on the sphere are generally larger compared to the ideal case, reaching up to 60° of direction error and 40° of position error (Fig. 5b; see the blue dots). However, these errors still remain well below the chance level, indicating that reasonable navigation on the sphere can be achieved using multiple planar maps.

## Discussion

To understand the nature of cognitive maps, we examined how people navigated a novel spherical environment. The path-integration task revealed a strong bias for planar geometry. Despite this bias, participants achieved a reasonable level of accuracy in the object-location memory test, potentially by utilizing multiple planar maps. In the following sections, we discuss our findings in relation to the existing literature, explore potential neural circuitry underlying the cognitive map, and speculate on the implications of multiple planar maps for solving broader cognitive tasks.

Our study highlights a pronounced planar geometry bias during path integration. This finding aligns with a recent study that also observed a dominance of Euclidean geometry on spherical and hyperbolic space (Widdowson & Wang, 2022). However, it is important to note several critical differences between this previous study and ours. In Widdowson and Wang, participants were immersed in a science-fiction-style warped universe, where light was curved and landmarks were absent. This means it was nearly impossible for participants to know whether they were walking in a curved world, potentially leading them to rely on familiar planar geometry for path integration. In contrast, the participants in our study were explicitly aware of the spherical surface on which they were walking. During the training phase, they encountered multiple landmarks along the great circle of the sphere. They also learned about the existence of multiple routes on the sphere. Half of participants even possessed prior knowledge of spherical geometry. Nevertheless, they all performed the path-integration task on the sphere as if they were on a plane, indicating a robust planar-geometry bias.

What could be the origin of this strong Euclidean bias in path integration? Is it hardwired in the brain, or acquired through experience or formal education? The philosopher Kant proposed that Euclidean geometry might be a priori knowledge in humans. Evidence from studies on individuals without formal math education, such as children and certain Amazonian indigenous groups, suggests an intuitive understanding of Euclidean geometrical concepts like line, right angle, and parallelism (Dehaene et al., 2006; Izard et al., 2011). In the natural world that humans live in, surfaces often have modest curvature that can be closely approximated by Euclidean geometry. The brain contains multiple specialized cell types that encode spatial information such as head direction, speed, and location—the basis of path integration (Moser et al., 2017; Wittmann & Schwegler, 1995). To test the impact of early geometrical experience on the development of spatial cells, Ulsaker-Janke et al., 2023, raised rat pups in an opaque spherical cage. Unlike the human participants who freely explored the entire spherical surface in our VR experiment, because of gravity the rat’s movement was constrained to the bottom part of the spherical cage. The spherical cage was different from a typical rectangular cage because it did not contain a vertical boundary that defines geometry. When the rats were later placed in a typical 2D environment, normal grid cells were absent on the first day but quickly emerged within a few days. These findings imply that a minimal experience with a typical environment is necessary for maturation of 2D grid fields that are anchored to an environment, but the topology of grid cells for a 2D flat surface is preconfigured independently of experience (Gardner et al., 2022; Ulsaker-Janke et al., 2023). Furthermore, studies on rodents walking on slopes or walls connected to a horizontal floor have shown that head-direction cells and grid cells continue to encode the direction and locations relative to a local surface, rather than a global 3D reference (Hayman et al., 2015; Stackman et al., 2000). This suggests that the brain treats joined surfaces as a Euclidean plane, resembling a hypothetical plane where multiple planar maps could be arranged in our simulation. Taken together, these findings suggest that the neural circuitry for path integration in the human brain may also be optimized for a flat surface, leading to the construction of multiple planar maps.

The dominance of the 2D path-integration system and the utilization of planar maps can be viewed as a useful feature rather than a limitation. Although path planning based on planar maps introduces some angular error at the start, our data and simulations have demonstrated that it is still possible to reach the target with a small overall error. This is because of the positive curvature of the sphere following Riemannian geometry. Building a precise map for high-dimensional space can be costly and inefficient when only a part of the space is relevant for behavior (e.g., walkable surfaces are 2D manifolds within a 3D world). If animals can achieve reasonable navigation accuracy by utilizing an existing path-integration system and multiple planar maps, there might be a limited demand for building more complex high-dimensional maps. It is also more ecological to use multiple small maps as opposed to building a single global map. Animals constantly interact with their environment and update their estimates of location and direction. Thus, a dynamic and flexible arrangement of cognitive maps becomes more desirable (Kim & Doeller, 2022).

The utilization of multiple planar maps on a sphere provides new insights into the longstanding question regarding the nature of cognitive maps. An earlier theory proposed a cognitive map that adheres to Euclidean geometry and contains precise metric information (O’Keefe & Nadel, 1978). However, it has been observed that people do not always rely on a coherent Euclidean map for navigation. Spatial memory and planning is strongly influenced by regional boundaries instead of Euclidean relations alone (Hirtle & Jonides, 1985; Kim & Maguire, 2018; Wiener & Mallot, 2003). Furthermore, in VR experiments in which the global adherence to Euclidean laws was broken through teleports, participants were still able to successfully navigate by attending to local spatial features (Warren et al., 2017). This suggests that cognitive maps may take on a graph or topographic form across multiple hierarchies (Chrastil & Warren, 2014; Peer et al., 2020). We propose that cognitive maps are Euclidean at the local level and that multiple planar maps are flexibly combined to represent a large and complex environment. This idea is in line with the neurophysiological mechanisms of grid cells, initially believed to provide global metric information but later proposed to encode spatial information rather locally (Ginosar et al., 2023). It is also noteworthy that simulations of grid cells predict imperfect global-scale path integration on the sphere (Stella et al., 2020).

Local planar maps might be useful for cognition beyond the physical navigation domain. There is abundant evidence that neural circuitry for physical navigation can be used for organizing various types of information, including odors and social and visual features (Aronov et al., 2017; Bao et al., 2019; Bellmund et al., 2018; Gärdenfors, 2000; Park et al., 2020). One limitation of the previous research is that the researchers used only 2D stimuli analogous to physical navigation on a flat surface. However, nonphysical information can be high-dimensional. Our findings of multiple planar maps for a spherical surface suggest that we can divide complex nonphysical space into multiple low-dimensional patches. The projection of multidimensional inputs onto low-dimensional vectors has been also proposed in the computational models of grid cells (Klukas et al., 2020).

We hope that some of the limitations in the current project can be addressed in future research. First, the vestibular and proprioceptive input in our setup was limited, because participants walked on a flat treadmill surface. It would be valuable to investigate whether additional 3D vestibular inputs, such as those experienced in a large microgravity environment or through the use of a 3D motion simulator (Barnett-Cowan et al., 2012), would result in the utilization of more volumetric cognitive maps. Second, exploring the impact of extended experience in nonflat environments on the format of cognitive maps would be intriguing. A recent experiment demonstrated that young children exhibited some understanding of a shortcut on spherical geometry (Huey et al., 2023). Investigating learning processes and the adaptability of humans to novel environments would contribute to a deeper understanding of cognitive-map formation.

## Supplemental Material

sj-docx-1-pss-10.1177_09567976241279291 – Supplemental material for Cognitive Maps for a Non-Euclidean Environment: Path Integration and Spatial Memory on a Sphere Supplemental material, sj-docx-1-pss-10.1177_09567976241279291 for Cognitive Maps for a Non-Euclidean Environment: Path Integration and Spatial Memory on a Sphere by Misun Kim and Christian F. Doeller in Psychological Science

## Transparency

*Action Editor:* Angela Lukowski

*Editor:* Patricia J. Bauer

*Author Contributions*

**Misun Kim:** Conceptualization; Data curation; Formal analysis; Investigation; Methodology; Project administration; Software; Supervision; Validation; Visualization; Writing – original draft; Writing – review & editing.

**Christian F. Doeller:** Conceptualization; Resources; Writing – review & editing.

**Open Practices:** The hypotheses and analysis plan were preregistered (https://doi.org/10.17605/OSF.IO/4TYM7) prior to data collection. Additional analyses were conducted for the triangle-completion task, as described in the Method section. Simulation of route planning was also added later. Materials, data, and analysis scripts are publicly available (https://doi.org/10.17605/OSF.IO/X4T9A). The authors are applying for a Computational Reproducibility Badge which will be awarded pending checks by the STAR team. This article has received the badges for Open Data, Open Materials, and Preregistration. More information about the Open Practices badges can be found at http://www.psychologicalscience.org/publications/badges.
