# Supplementary material for: Cognitive Maps for a Non-Euclidean Environment: Path Integration and Spatial Memory on a Sphere
Source: Psychol Sci. 2024 Oct 25;35(11):1217–30. doi: 10.1177/09567976241279291 (PMC13020992; doi:10.1177/09567976241279291)

# Supplementary Materials

Supplementary videos can be found in OSF repository, which also contains raw data and analysis scripts. A link and caption for each video is shown below.

**Supplementary Video 1**. A learning phase on the sphere. Participants freely explore the environment to learn the location of 12 landmark animals. A participant’s first-person perspective view is shown on the left side. The right side shows the bird’s eye view of the environment and the location of a participant (outlined as a white box). Of note, a field of view was larger and depth perception was better when wearing the VR goggles in the actual experiment.

<https://osf.io/2es6n/>

**Supplementary Video 2**. A learning phase on the plane. Participants could only see the short distance because of the fog we added to match the visibility between the plane and sphere.

<https://osf.io/jpm5a>

**Supplementary video 3**. An object-location test phase on the sphere.

<https://osf.io/aux7s/>

**Supplementary video 4**. An object-location test phase on the plane.

<https://osf.io/nw3mc/>

**Supplementary video 5**. A triangle completion task on the sphere.

<https://osf.io/ks639/>

**Supplementary video 6**. A triangle completion task on the plane.

<https://osf.io/y75nu/>

## Supplementary Figure 1


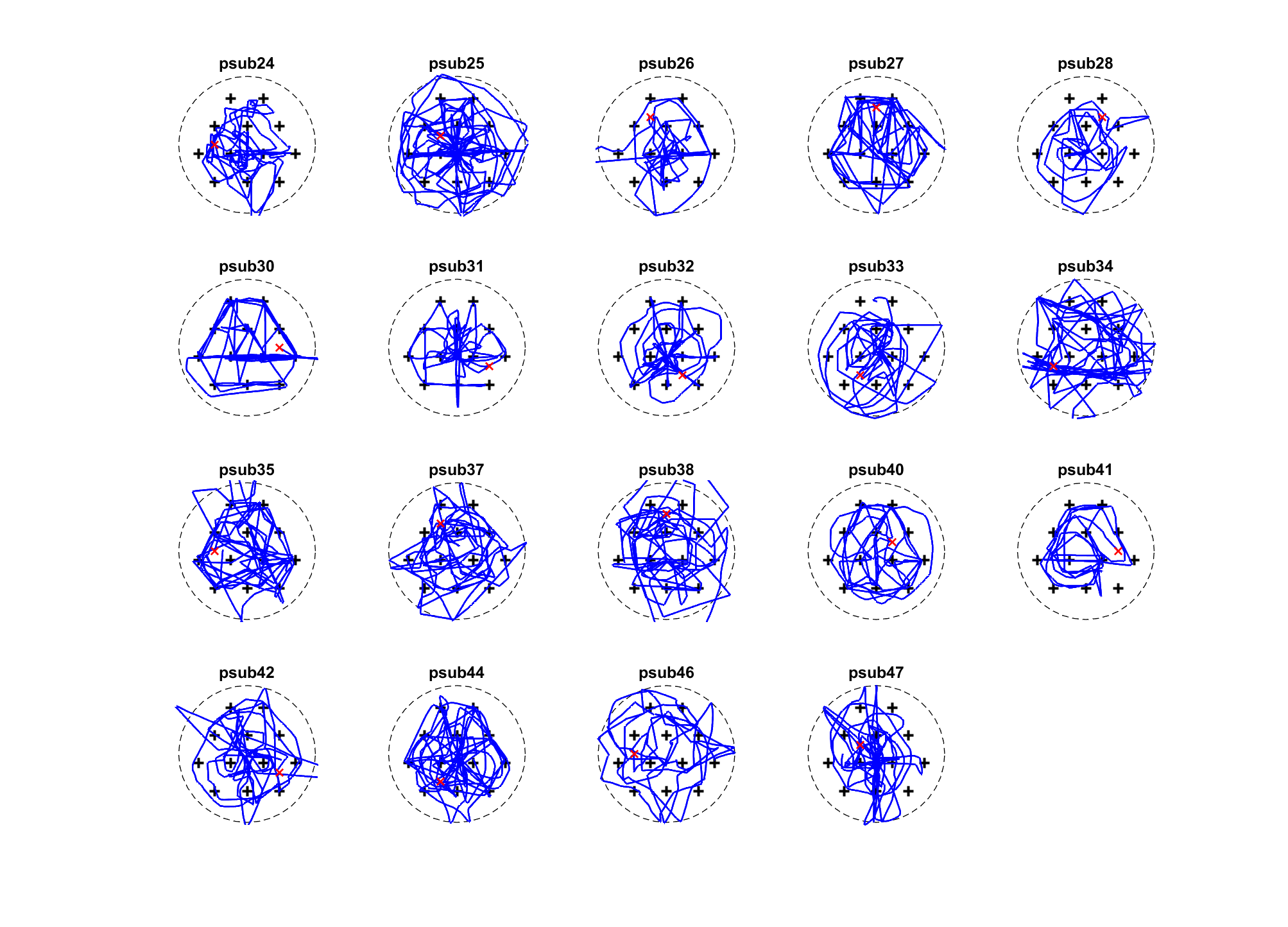


**Supplementary Figure 1**. All participants’ trajectories (blue lines) during the free exploration phase in the plane condition are shown. An invisible boundary that prevented participants from moving too far away from the landmarks (black crosses) is shown as a dashed circle. Most participants stayed near the center of the environment, rather than repeatedly approaching the invisible wall. Red crosses show the start location of each participant.

## Supplementary Figure 2


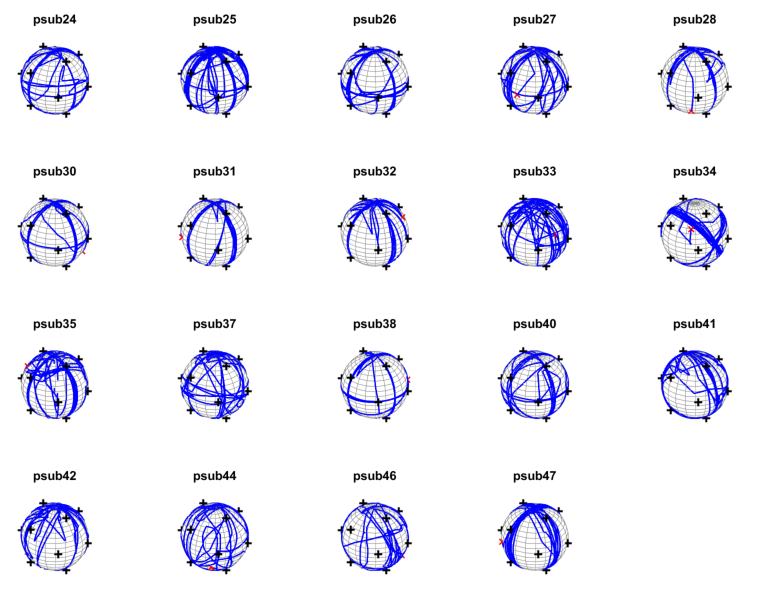


**Supplementary Figure 2**. All participant’s trajectory (blue lines) during the free exploration phase in the sphere condition are shown. Most participants repeatedly moved between the landmarks (black crosses) and followed a great circle on the sphere. Red cross shows the start location of each participant.

## Supplementary Figure 3


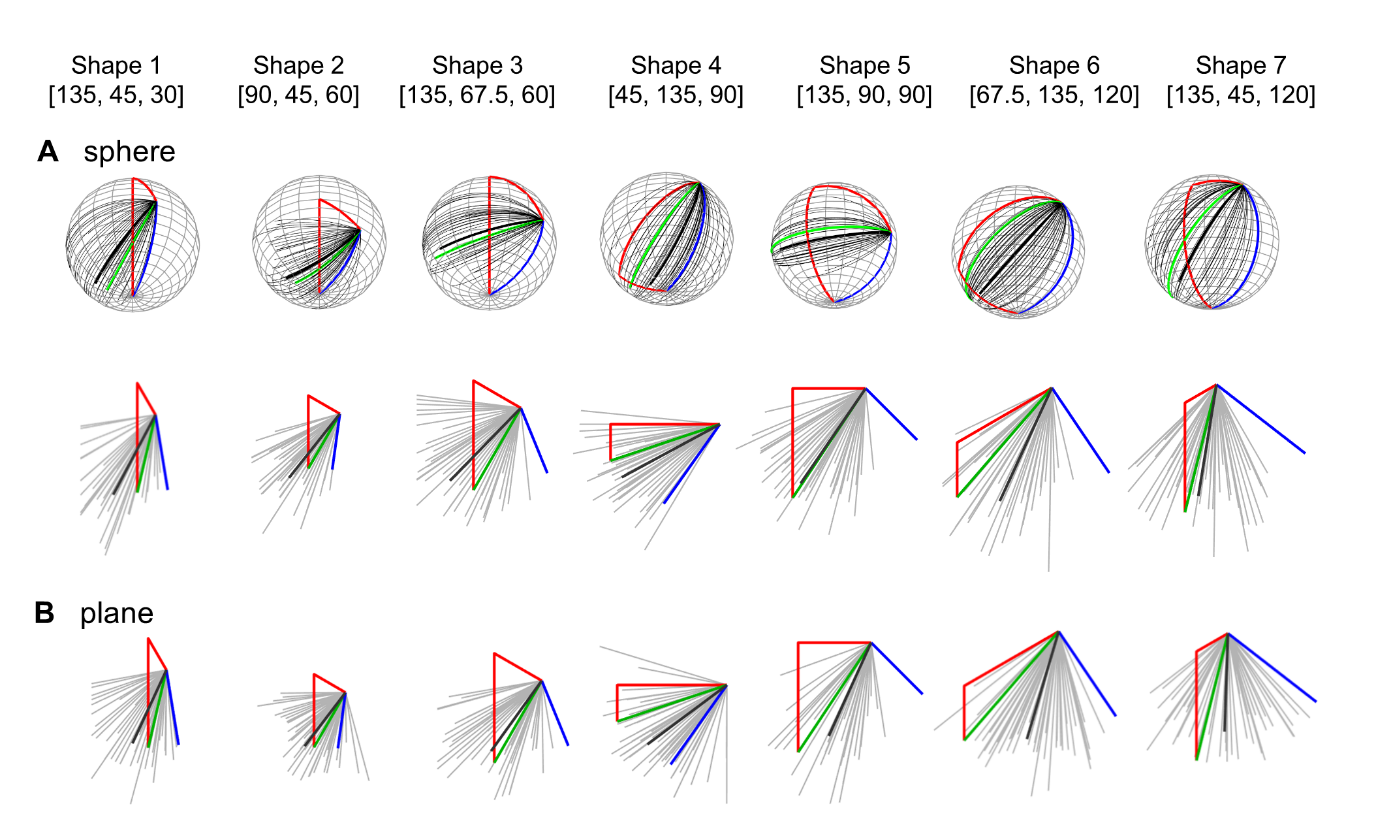


**Supplementary Figure 3**. All triangle completion task data. **A**. Results on the sphere are presented in 3D views (top row) and a 2D projected view (bottom row). **B**. Results on the plane. Red lines, outbound path; thick black line, mean inbound path; grey line, inbound path of individual trials; green line, ideal inbound path on the plane; blue line, ideal inbound path on the sphere. Shape parameters for each triangle are shown on top, [length of the first outbound leg, length of the second leg, inner angle between the first and second leg]. The unit is in degree.

## Supplementary Figure 4

**
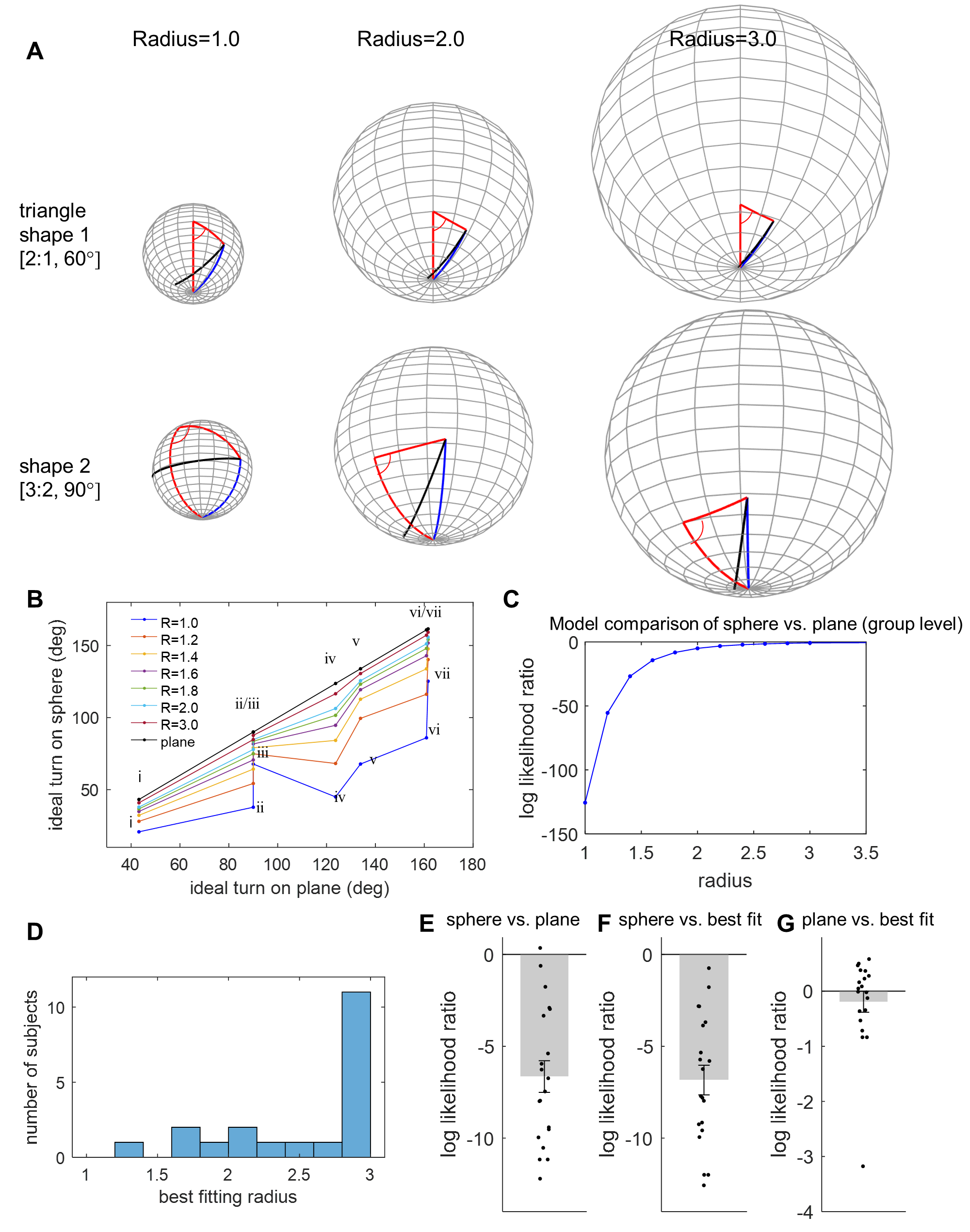
**

**Supplementary Figure 4**. Spherical models with different radii. **A**. Two triangle shapes used in the experiment [the ratio of the first : second leg length, the inner angle between the legs] are overlaid on spheres of different radii. When a radius is small, ideal inbound paths on the sphere (blue line) deviate substantially from the ideal inbound paths on the plane (black line). As the radius increases, the spherical triangle becomes flatter and the spherical inbound path becomes indistinguishable from the planar inbound path. **B**. Ideal turn angles for the seven triangle shapes on the spheres of different radii. In the original radius (R=1.0, blue line), ideal turn angles on the sphere are clearly distinguished from the turn angles on the plane (black line). We selected these triangle shapes and radius to minimize the correlation between the sphere and plane model. For instance, the turn angle for the shape ii and iii are identical and smaller than the shape iv on the plane (black line), whereas the turn angle for the shape iii is greater than ii and iv on the sphere (blue line). As the radius increases, turn angles on the sphere converge quickly to the plane model. At R=1.6 (purple line), turn angles already show a monotonical pattern similar to the plane model. At R=3.0 (dark red line), turn angles are almost indistinguishable from the plane model (black line). **C**. Log likelihood ratios of the sphere model of various radii relative to the plane model at the group level. When a radius is small, a sphere model fits worse than a plane model. As the radius increases, the sphere model fits the data as good as the plane model. **D**. A distribution of best-fitting radius of individual subject data. The largest radius model (=effectively planar) was the best model for more than half of the participants. **E**. Log likelihood ratios of a veridical sphere model (radius=1) compared to the plane model for each participant. **F**. Log likelihood ratios of a veridical sphere model (radius=1) compared to the best fitting radius model for each participant. **G.** Log likelihood ratios of a plane model (radius=1) compared to the best fitting radius model for each participant.

## Supplementary Figure 5


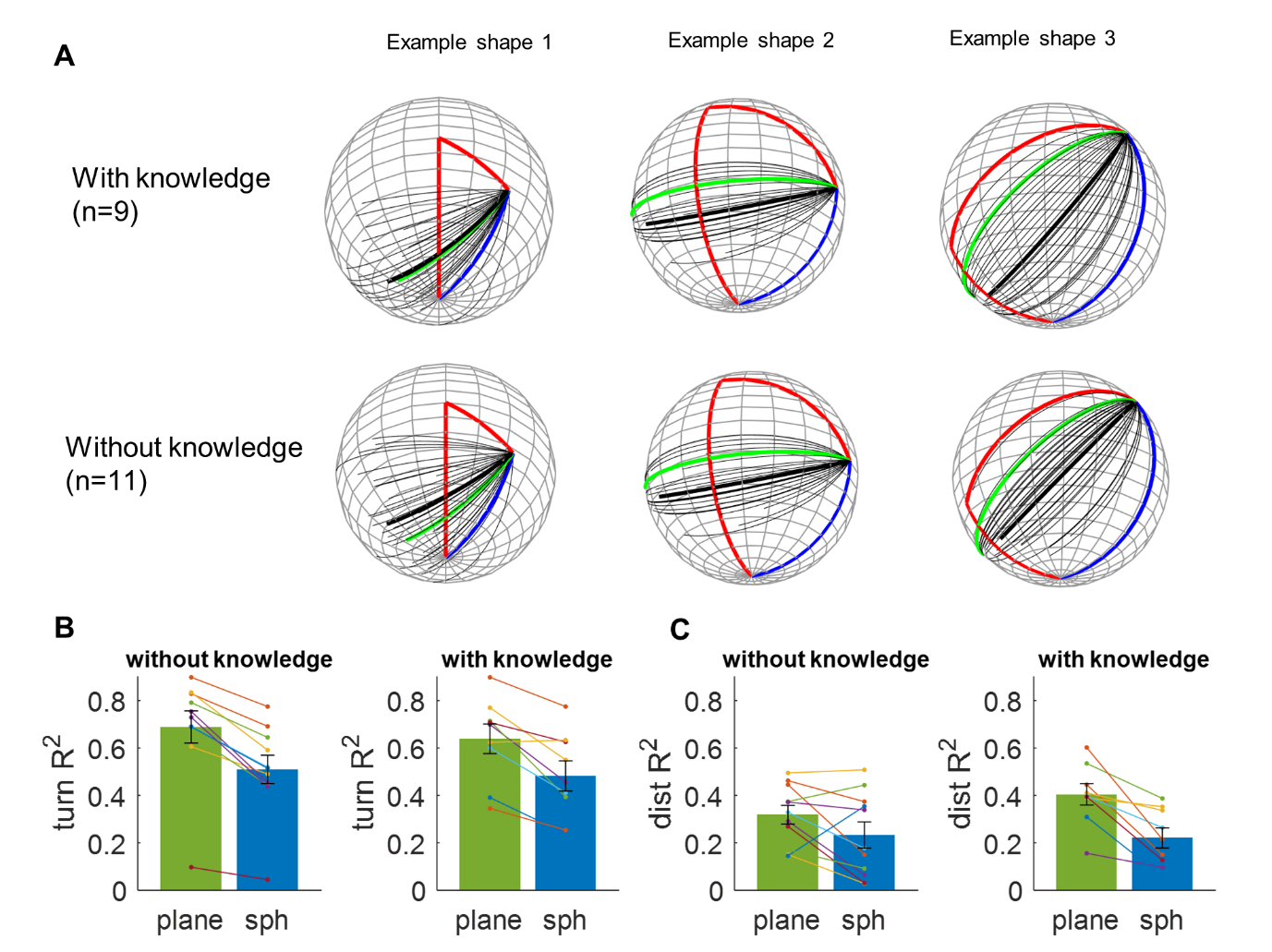


**Supplementary Figure 5**. Triangle completion task result shown separately for those with prior knowledge on spherical geometry and those without. Both groups show a strong planar bias (Bayes factor for the planar vs. spherical model, turn angle=7.2e20, distance=2.9e14 in participants with knowledge; turn angle=4.6e33, distance=2.7e8 in participants without knowledge) **A**. Individual trial and mean responses are shown for three example shapes. Red lines, outbound path; thick black line, mean inbound path; grey line, inbound path of individual trials; green line, ideal inbound path on the plane; blue line, ideal inbound path on the sphere. **B**. Model fit for the turn angle by a plane and sphere model at individual participant level. Error bar is the standard error. **C**, Same as B for the inbound distance.

## Supplementary Figure 6


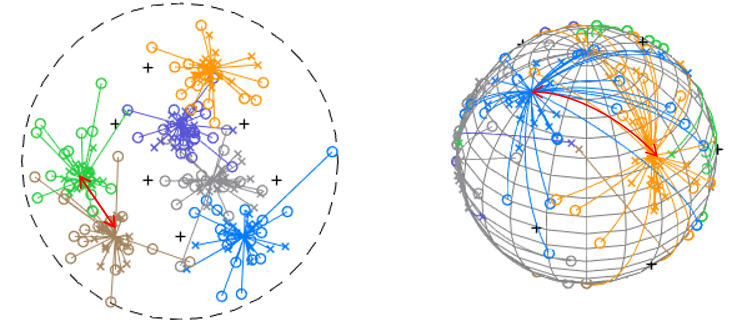


**Supplementary Figure 6**. Object-location memory test result on the plane and on the sphere. Recalled object locations in the last trials of all participants (colored ‘o’ and ‘x’) are overlaid. Lines connecting the individual recalled location and the corresponding target location are displayed to help readers to estimate the size of the positional error. Position errors were larger for long distance trials (‘o’) than short distance trials (‘x’). Geodesic distance between the neighboring landmarks (line with red arrows) were identical on the plane and sphere condition (~63 deg).

## Supplementary Figure 7


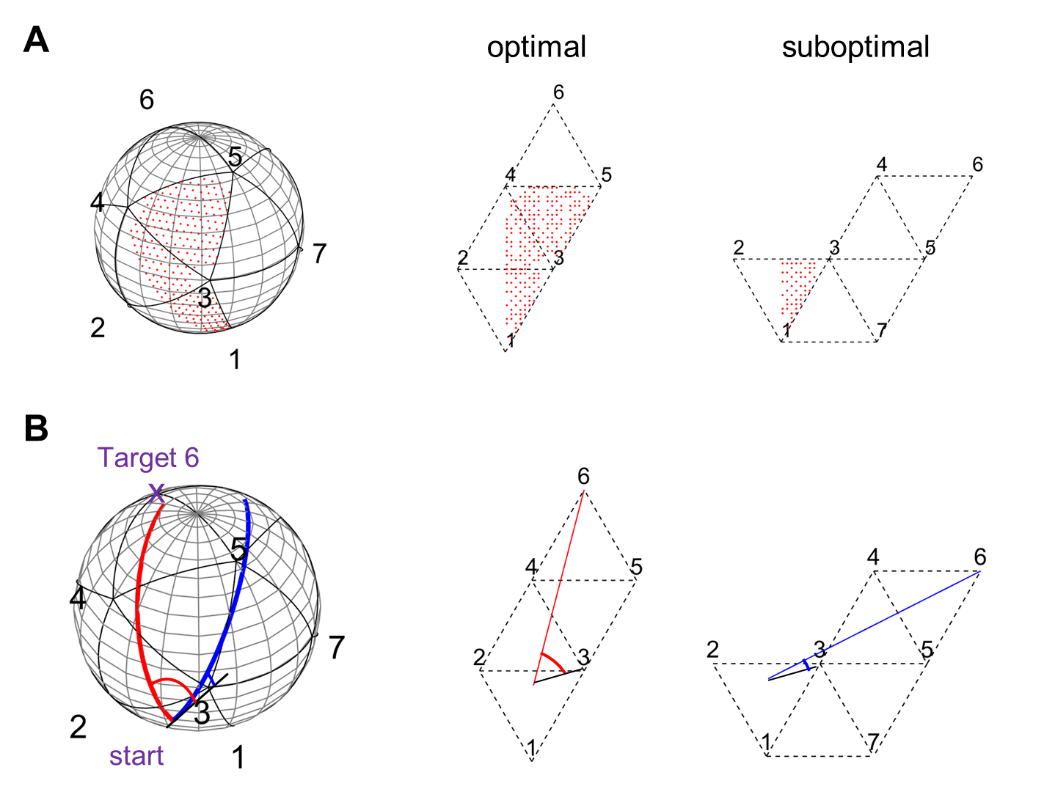


**Supplementary Figure 7**. Simulation of route planning when a spherical surface is approximated by multiple triangular patches defined by landmarks. **A**. Red dots indicate random start locations of varying distances from a target landmark 6. **B**. Example paths from a start located between the landmarks 1,2, and 3 to the target landmark 6. A path distance and direction relative to a reference landmark (e.g. landmark 3 in this example) is measured on a plane and applied on a sphere. The end of the paths planned on the planar maps are in close proximity to the true target location on the sphere. Red line, a path based on an optimal arrangement of planar map; blue line, a path based on a suboptimal arrangement; x, a target on the sphere.

## Supplementary Table 1.

A linear mixed model for the turn angle on the sphere with a planar geometry predictor


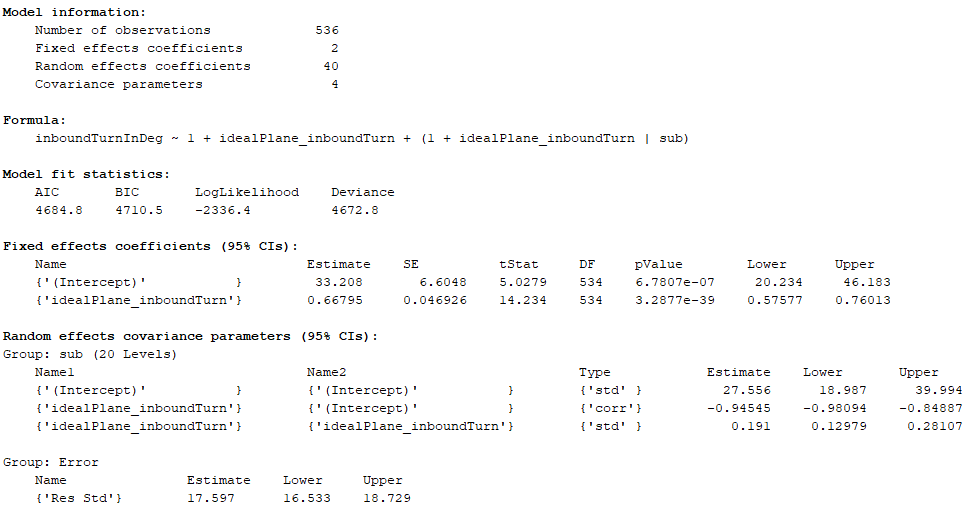


## Supplementary Table 2.

A linear mixed model for the turn angle on the sphere with a spherical geometry predictor


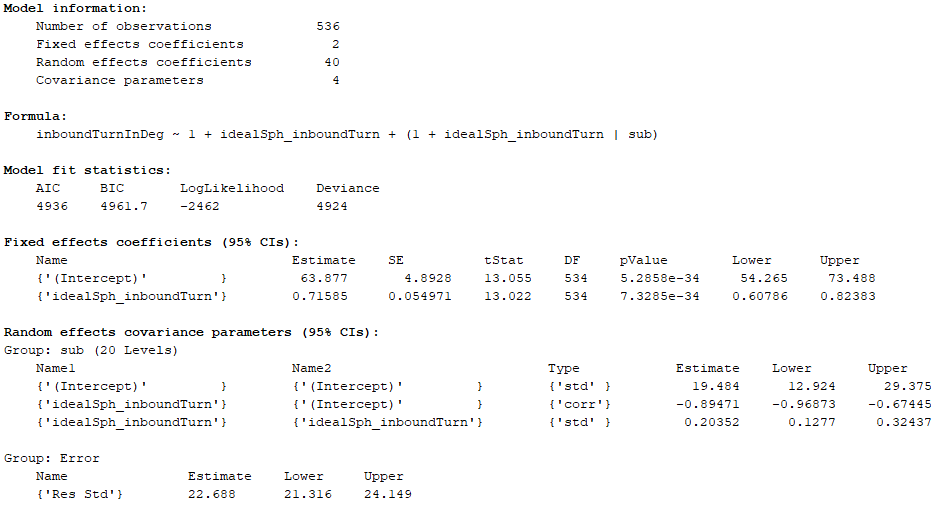


## Supplementary Table 3.

A linear mixed model for the distance on the sphere with a planar geometry predictor


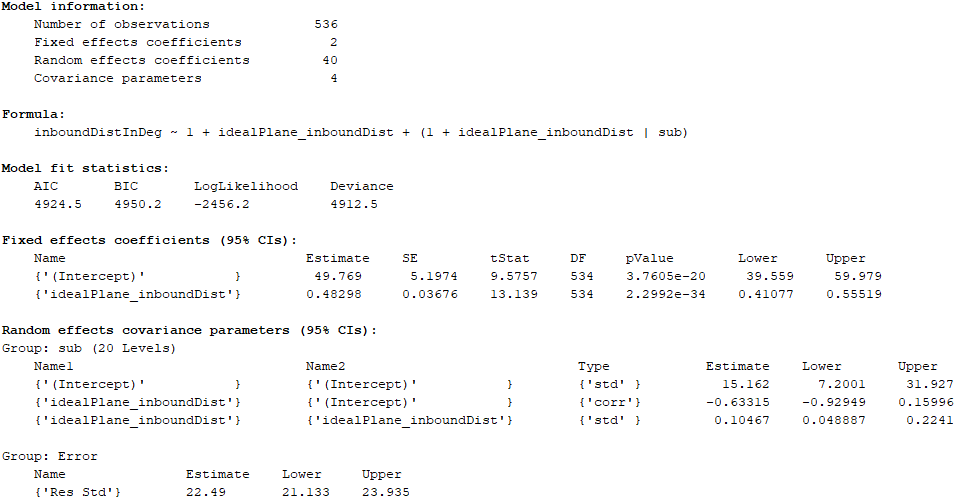


## Supplementary Table 4.

A linear mixed model on the distance on the sphere with a spherical geometry predictor


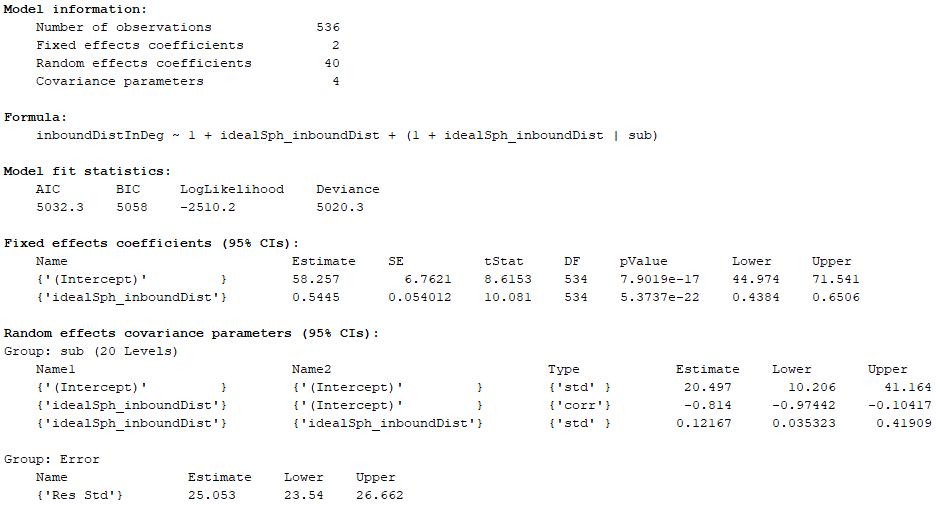

Supplement: sj-docx-1-pss-10.1177_09567976241279291 – Supplemental material for Cognitive Maps for a Non-Euclidean Environment: Path Integration and Spatial Memory on a Sphere [file sj-docx-1-pss-10.1177_09567976241279291.docx]
